# GADD45A and GADD45B as Novel Biomarkers Associated with Chromatin Regulators in Renal Ischemia-Reperfusion Injury

**DOI:** 10.3390/ijms241411304

**Published:** 2023-07-11

**Authors:** Ming Xie, Ruiyan Xie, Pengcheng Huang, Desmond Y. H. Yap, Peng Wu

**Affiliations:** 1 Department of Urology, Nanfang Hospital, Southern Medical University, Guangzhou 510515, China; 2 Division of Nephrology, Department of Medicine, Queen Mary Hospital, The University of Hong Kong, Hong Kong 999077, China

**Keywords:** renal ischemia-reperfusion injury, chromatin regulators, renal IRI mice model, network, biomarkers, DNA damage-inducible protein 45, *GADD45A*, *GADD45B*, immune cells, plasmacytoid dendritic cells

## Abstract

Chromatin regulators (CRs) are essential upstream regulatory factors of epigenetic modification. The role of CRs in the pathogenesis of renal ischemia-reperfusion injury (IRI) remains unclear. We analyzed a bioinformatic analysis on the differentially expressed chromatin regulator genes in renal IRI patients using data from public domains. The hub CRs identified were used to develop a risk prediction model for renal IRI, and their expressions were also validated using Western blot, qRT-PCR, and immunohistochemistry in a murine renal IRI model. We also examined the relationships between hub CRs and infiltrating immune cells in renal IRI and used network analysis to explore drugs that target hub CRs and their relevant downstream microRNAs. The results of machine learning methods showed that five genes (*DUSP1*, *GADD45A*, *GADD45B*, *GADD45G*, *HSPA1A*) were upregulated in renal IRI, with key roles in the cell cycle, p38 MAPK signaling pathway, p53 signaling pathway, FoxO signaling pathway, and NF-κB signaling pathway. Two genes from the network, *GADD45A* and *GADD45B* (growth arrest and DNA damage-inducible protein 45 alpha and beta), were chosen for the renal IRI risk prediction model. They all showed good performance in the testing and validation cohorts. Mice with renal IRI showed significantly upregulated *GADD45A* and *GADD45B* expression within kidneys compared to sham-operated mice. *GADD45A* and *GADD45B* showed correlations with plasmacytoid dendritic cells (pDCs) in infiltrating immune cell analysis and enrichment in the MAPK pathway based on the weighted gene co-expression network analysis (WGCNA) method. Candidate drugs that target *GADD45A* and *GADD45B* include beta-escin, sertraline, primaquine, pimozide, and azacyclonol. The dysregulation of *GADD45A* and *GADD45B* is related to renal IRI and the infiltration of pDCs, and drugs that target *GADD45A* and *GADD45B* may have therapeutic potential for renal IRI.

## 1. Introduction

Ischemia-reperfusion injury (IRI) is a pathophysiological condition characterized by transient reduction or discontinuation in perfusion to an organ followed by the restoration of blood supply and concomitant reoxygenation [1]. Renal IRI is an important pathophysiological process in various forms of acute kidney injury (AKI), including shock [2], severe sepsis [3], major cardiothoracic surgery [4], trauma [2], and kidney transplantation [2]. Notably, the presence of AKI is a robust predictor of unfavorable outcomes in shock [3] and major operations [5]. In the context of kidney transplantation, renal IRI is inevitable during the surgical procedure and is associated with delayed graft function, early renal allograft failure and rejection, increased renal fibrosis of transplanted kidney, and reduced long-term allograft survival [6,7]. However, there were no proven effective therapies to prevent or attenuate renal IRI. An improved understanding of the pathogenesis of IRI may therefore potentially help devise novel strategies to avoid or mitigate renal IRI, thereby improving the overall clinical outcomes in patients with AKI or kidney transplantation. 

The pathophysiological mechanisms for renal IRI were highly complex. Putative molecular and cellular events include exaggerated innate and adaptive immune responses, increased oxidative stress [8], necroptosis and ferroptosis [7], DNA damage [9], mitochondrial dysfunction [10], and enhanced apoptosis [11]. Emerging data have suggested that epigenetic regulation, a complex process of heritable changes in gene expression without alteration in the DNA sequences, plays an instrumental role in renal IRI and kidney transplantation [12]. Chromatin regulators (CRs) are essential upstream regulatory factors of epigenetic modification and can be classified into three major types, namely the DNA methylators, histone modifiers, and chromatin remodelers [13]. 

The dysregulation of several CRs has been associated with renal IRI [14,15,16,17]. For instance, peptidyl arginine deiminase-4 (PAD4), which converts arginine residues of histones to citrulline [14], is elevated in renal tubular cells after renal IRI, and PAD4-deficient mice are protected against renal IRI [15]. The enhancer of zeste homolog 2 (EZH2), a methyltransferase of histone H3 lysine 27, is upregulated in the progression of renal IRI, while the inhibition of EZH2 by its inhibitor DZNeP alleviates kidney injury by blocking reactive oxygen species generation via the ALK5/Smad2/3 axis [16]. G9a, a H3K9 methyltransferase of histone, was reported to induce renal IRI by forming a functional transcription repressor complex with chromobox homolog 1 (CBX1) on the Sirt1 promoter [17]. Furthermore, the aberrant regulation of CRs was also observed in chronic kidney disease (CKD) [18], autosomal dominant polycystic kidney disease [19], and kidney injury induced by cisplatin toxicity or unilateral ureteral obstruction [20]. The exact role of CRs in renal IRI, nonetheless, remains elusive and has not been systematically studied. The involvement of the innate and adaptive immune responses is also well recognized in renal IRI. The activation and recruitment of different immune cells, including renal dendritic cells (DC), macrophages, neutrophils, natural killer cells, and T and B lymphocytes, have crucial functions in mediating the acute insult and repair of renal IRI [21]. While the immune response assumes key pathogenic significance in renal IRI, there are limited data regarding the interaction between CRs and immune-reactive cells.

Based on these backgrounds, we conducted a bioinformatic analysis to identify CRs related to renal IRI and developed a risk prediction model based on these CRs. The pathogenic relevance of these CRs was also validated by Western blot, qRT-PCR, immunohistochemistry, and immunofluorescence staining in a murine renal IRI model. We further examined the relationships between these hub CRs and immune-reactive cells and investigated potential medications that can attenuate these hub CRs.

## 2. Results

### 2.1. Identification and Enrichment Analysis of CRs-Associated DEGs in Renal Ischemia-Reperfusion Injury Using Three Machine Learning Models

**Figure 1:**
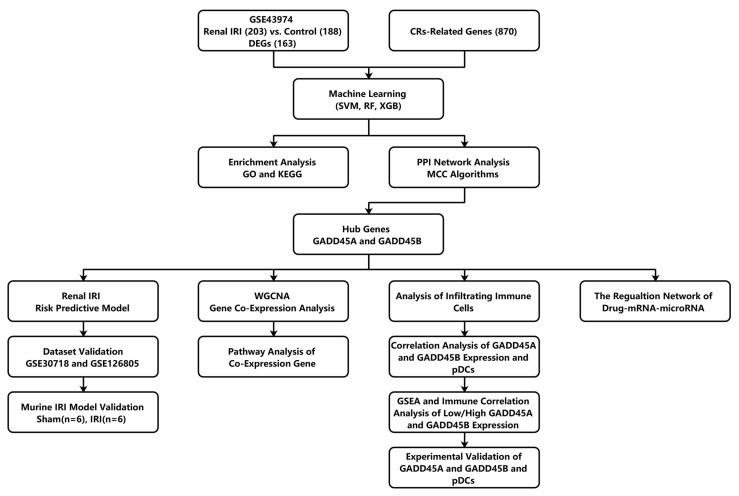
Flow chart.

**Figure 2:**
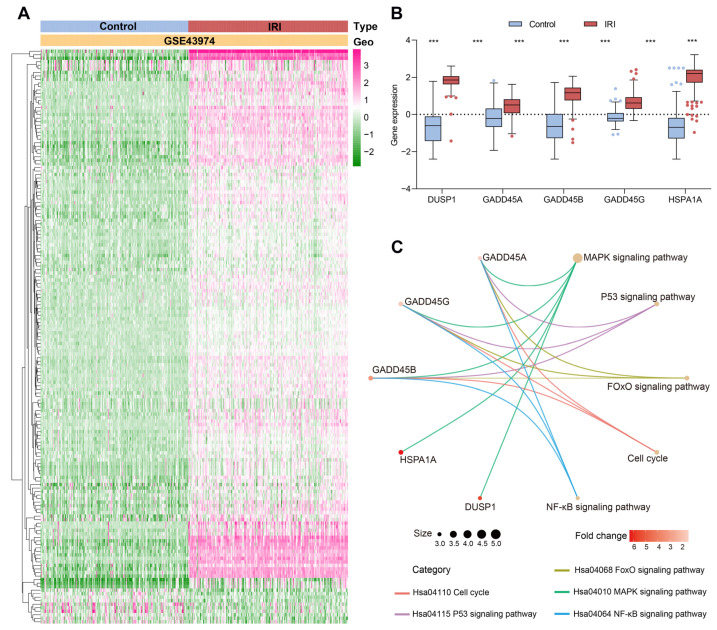
Functional enrichment analysis of the chromatin regulator (CR)-related differentially expressed genes (DEGs) in the human renal ischemia-reperfusion injury (IRI) GSE43974 dataset based on three machine learning methods. (**A**) Heatmap showing DEGs of human renal IRI. (**B**) Five transcriptional expression levels of CR-related intersection genes. (**C**) Kyoto Encyclopedia of Genes and Genomes enrichment analysis of five CR-associated intersected DEGs. *** *p* < 0.001.

The flow chart of this study is shown in Figure 1. A total of 163 DEGs were identified in human renal IRI samples, with 153 overexpressed genes and 10 downregulated genes (Figure 2A). We further analyzed CR-related DEG genes using three machine learning models, SVM (support vector machine) [22], RF (random forest) [23], and XGB (extreme gradient boosting) [24]. To estimate these models, reverse cumulative distributions of residuals were created to select candidate CRs (Figure S1A). As shown in Figure S1B, the SVM model showed the smallest value of the residuals, indicating the least difference between the observed value and the model estimate compared to the RF and XGB models. The SVM model also showed the best accuracy value (AUC ROC was 0.987), while RF and XGB machine learning models showed a similar degree of accuracy (AUCs ROC were 0.984 and 0.984, respectively). The five intersection genes identified by three machine learning methods were selected. These five intersection genes (*DUSP1*, *GADD45A*, *GADD45B*, *GADD45G*, and *HSPA1A*) were all significantly upregulated compared to the control samples (*p* < 0.05; Figure 2B).

The GO enrichment analysis showed that these five intersected genes were enriched to 24 significant ontology terms. The top five most significant BP pathways included the regulation of the p38 MAPK cascade (GO:1900744), the p38 MAPK cascade (GO:0038066), the positive regulation of the p38 MAPK cascade (GO:1900745), the regulation of the stress-activated MAPK cascade (GO:0032872), and the regulation of the stress-activated MAPK cascade (GO:0070302). The intersection genes of the top BP terms were *GADD45A*, *GADD45B*, and *GADD45G*, which were all enriched in the MAPK-related pathway. The only CC term was nuclear speck (GO:0016607), and the MF term was protein N-terminus binding (GO:0047485) (Table 1). KEGG analysis showed that the five most significant signaling pathways were the MAPK signaling pathway (*p* = 3.45 × 10^−7^), p53 signaling pathway (*p* = 1.36 × 10^−5^), cell cycle (*p* = 6.99 × 10^−5^), FoxO signaling pathway (*p* = 7.85 × 10^−5^), and NF-κB signaling pathway (*p* = 3.93 × 10^−5^) (Figure 2C).

### 2.2. Construction of a CRs-Associated Predictive Nomogram and Validation

**Figure 3:**
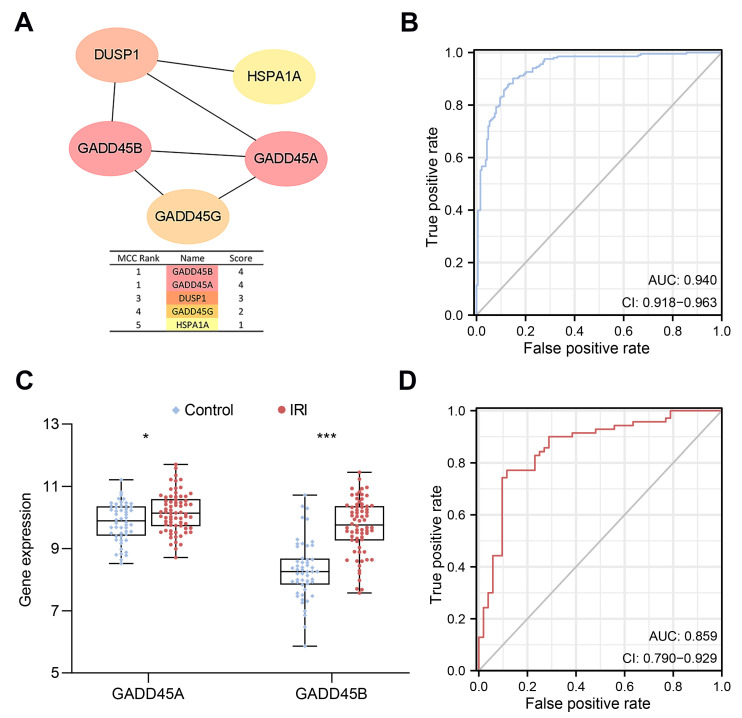
The construction and validation of a chromatin regulator (CR)-associated predictive human renal ischemia-reperfusion injury (IRI) risk score nomogram. (**A**) The protein–protein interaction (PPI) network of the five intersection genes. (**B**) Receiver operator characteristic (ROC) curve of the predictive IRI risk model in the training cohort. (**C**) Validation of *GADD45A* and *GADD45B* expression and (**D**) the predictive IRI risk model in the merged datasets of human GSE30718 and GSE126805 (validation cohort). *GADD45A* (growth arrest and DNA damage inducible protein 45 alpha); *GADD45B* (growth arrest and DNA damage inducible protein 45 beta). * *p* < 0.05, *** *p* < 0.001.

The cytoHubba plugin [25], a cytoscape plugin, was performed to select the hub nodes in the PPI network. MCC (maximal clique centrality) [25] algorithms were used to analyze the important modules [25]. Both *GADD45A* and *GADD45B* showed the highest scores and were further selected as the hub genes for the subsequent analysis (Figure 3A).

A CR-associated nomogram was constructed using hub genes to predict the risk of renal IRI (Figure S2A). Renal IRI risk score = (1.2387 × expression value of *GADD45A*) + (3.815 × expression value of *GADD45B*). Renal IRI kidney tissue samples were divided into high- and low-expression groups based on the cut-off of the median gene expression. The nomogram calibration curve showed an acceptable agreement between prediction and actual observation in the training cohort (mean absolute error = 0.012, Figure S2B). The risk score model showed excellent predictive value for renal IRI. The area under curve (AUC) of ROC was 0.940 (Figure 3B). 

We further verified our risk model in the validation cohort using data merged from two separate human datasets (GSE30718 and GSE126805). Similarly, *GADD45A* and *GADD45B* in renal IRI samples were both significantly upregulated compared to the control samples (Figure 3C), and the risk score model showed good performance for predicting renal IRI (AUC of the ROC was 0.859) (Figure 3D).

### 2.3. Validation of GADD45A and GADD45B Expression in Mice Renal IRI Model

**Figure 4:**
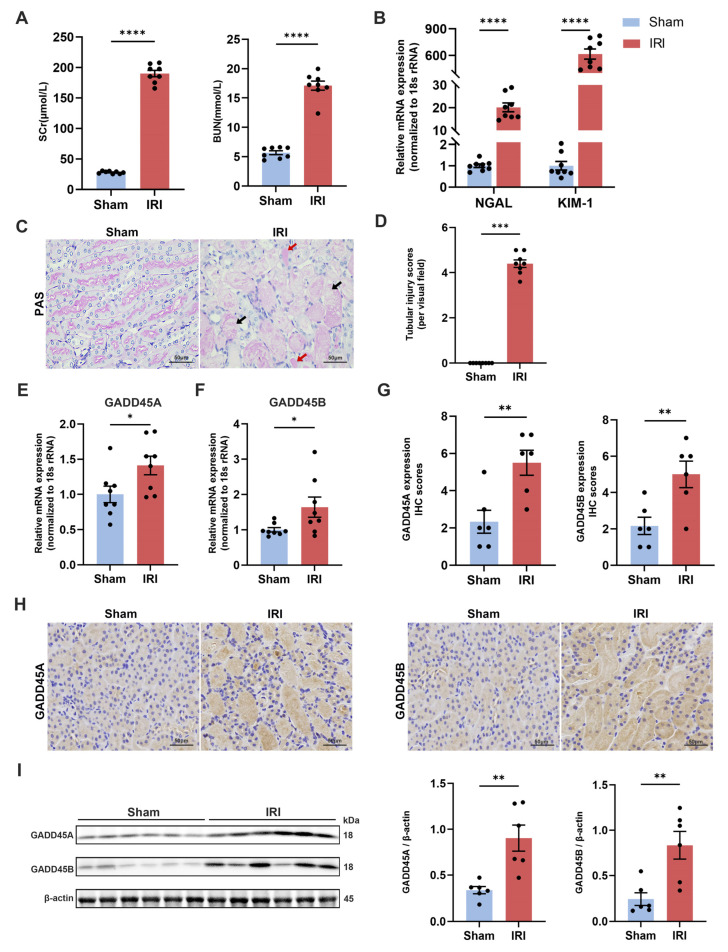
The validation of bioinformatics data using a murine renal ischemia-reperfusion injury (IRI) model. Levels of (**A**) SCr and BUN, (**B**) *NGAL* and *KIM-1* mRNA expression within kidneys in IRI and sham-operated mice, n = 8 per group. (**C**) Representative photos (400×) of PAS staining and (**D**) the quantitative analysis of the tubular damage of renal tissue. The photomicrographs of the IRI group showed the disappearance of the brush border, the necrosis and detachment of tubular epithelial cells (black arrow), and the formation of protein cast (red arrow), n = 8 per group. (**E**) *GADD45A* and (**F**) *GADD45B* transcriptional levels in kidney tissues of renal IRI and sham-operated mice, n = 8 per group. (**G**) The statistical analysis of the GADD45A and GADD45B protein expression levels in immunohistochemical (IHC) staining, n = 6 per group. (**H**) Representative images of the IHC staining of GADD45A and GADD45B in renal IRI and sham-operated mice, n = 6 per group. (**I**) Western Blot analysis of GADD45A and GADD45B relative protein expression in renal tissue lysates from IRI and sham-operated groups, respectively, followed by the statistical analysis of the ratio of band densities of GADD45A and GADD45B to β-actin, n = 6 per group. * *p* < 0.05, ** *p* < 0.01, *** *p* < 0.001, **** *p* < 0.0001. Scale bars (black), 50 μm. SCr, serum creatinine; BUN, blood urea nitrogen; PAS, periodic acid-Schiff; *NGAL*, neutrophil gelatinase-associated lipocalin; *KIM-1*, kidney injury molecule-1.

We used a murine renal IRI model to further evaluate the expression of *GADD45A* and *GADD45B* in renal tissue. SCr (serum creatinine) and BUN (blood urea nitrogen) in the renal IRI group were significantly higher than the sham-operated group (190.10 ± 5.28 μmol/L vs. 27.88 ± 0.71 μmol/L; and 17.10 ± 0.79 mmol/L vs. 5.67 ± 0.33 mmol/L, *p* < 0.0001 and *p* < 0.0001, respectively) (Figure 4A). The renal IRI group also showed the increased mRNA expression of renal injury markers neutrophil gelatinase-associated lipocalin (*NGAL*) and kidney injury molecule-1 (*KIM-1*) compared to control mice (Figure 4B) (relative quantity (RQ) 20.16 ± 1.96 vs. 1.00 ± 0.08 and 615.58 ± 57.21 vs. 1.00 ± 0.20, *p* < 0.0001 and *p* < 0.0001, respectively). The renal IRI mice demonstrated prominent histological features of renal tubular injury, including necrosis and the detachment of tubular epithelial cells, the formation of protein cast, and the disappearance of the brush border (Figure 4C,D). The IRI group showed significantly upregulated *GADD45A* and *GADD45B* mRNA expression compared to sham-operated mice (RQ 1.41 ± 0.13 vs. 1.00 ± 0.12 and 1.64 ± 0.28 vs. 1.00 ± 0.06, *p* < 0.05 and *p* < 0.05, respectively, Figure 4E,F). The IHC staining showed increased protein intensity of GADD45A and GADD45B (Figure 4G,H) in renal IRI mice kidney tissues compared to sham-operated animals. Increased protein expression levels of GADD45A and GADD45B were also confirmed by Western blot analysis (Figure 4I).

### 2.4. Identification of GADD45A and GADD45B Co-Expression Genes Network in Renal Ischemia-Reperfusion Injury Using WGCNA

**Figure 5:**
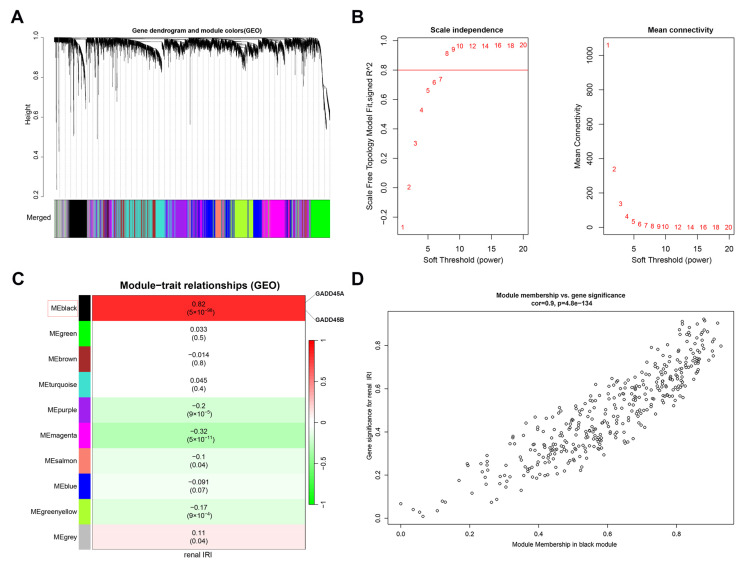
The construction of *GADD45A*, *GADD45B*, and the co-expression gene network using weighted gene co-expression network analysis (WGCNA) in renal ischemia-reperfusion injury (IRI). (**A**) A merged dendrogram of gene co-expression modules. (**B**) The analysis of scale independence and mean connectivity with soft-thresholding powers (β). (**C**) The heatmap represents the correlation of different color modules with renal IRI. The black module showed the most significant association with renal IRI, including *GADD45A* and *GADD45B*. (**D**) Scatterplot showed the correlation of module membership from the black module and gene significance for the renal IRI.

Genes with the largest variance in the top 25% (5188 genes) of all the genes in the dataset were further analyzed. The threshold of 0.25 was first used to merge the similar-trait-associated genes (Figure 5A). We selected β = 8 (scale free R2 = 0.85) as the soft threshold (Figure 5B). The gene expression matrix was divided into ten modules (Figure 5C). Each module contained at least 50 genes expressing similar traits. The most significant co-expressed module with *GADD45A* and *GADD45B* was the MEblack section, which included 368 genes (correlation coefficient = 0.82, *p* = 5 × 10^−98^, Figure 5C). The 368 co-expression genes showed a positive correlation with renal IRI (correlation coefficient = 0.9, *p* = 4.8 × 10^−134^, Figure 5D). 

### 2.5. Pathway Analysis of GADD45A and GADD45B Co-Expression Genes Network in Renal Ischemia-Reperfusion Injury

**Figure 6:**
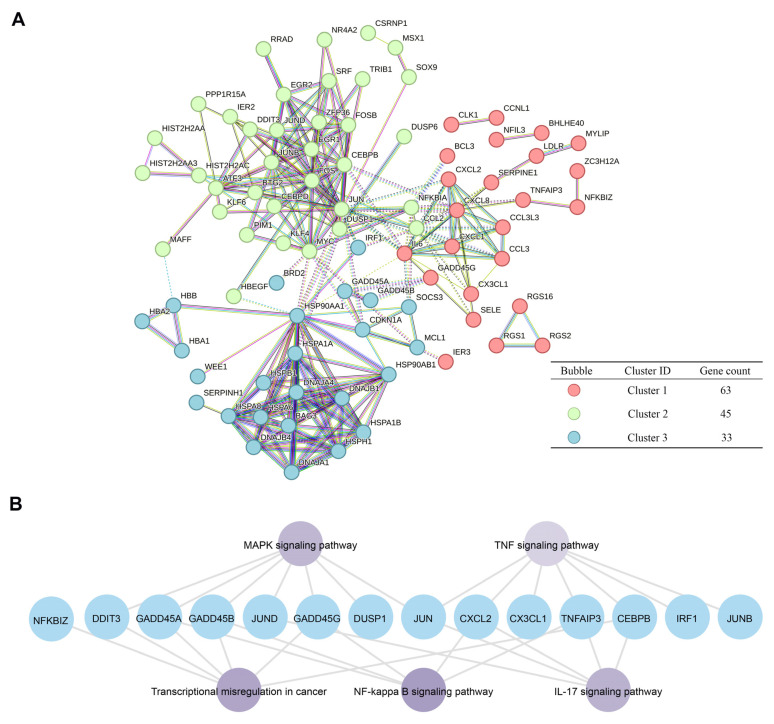
The pathway analysis of *GADD45A*, *GADD45B*, and co-expression genes in renal ischemia-reperfusion injury (IRI). (**A**) Three clusters in the protein–protein interaction network. The corresponding proteins of intersected genes were divided into three clusters in a protein–protein interaction network using the k-means method. (**B**) The KEGG pathways analysis of cluster 1 co-expression genes, including *GADD45A* and *GADD45B*.

A total of 152 intersection genes of 368 co-expression genes selected by WGCNA (weighted gene co-expression network analysis) [26] and 163 DEGs in renal IRI were selected (Supplemental Spreadsheet S1). The network of 152 intersection genes was constructed using the Enrichr online tool [27]. The 141 corresponding proteins were divided into three clusters in a PPI network, containing 63, 45, and 33 proteins, respectively, according to k-means clustering analysis (Figure 6A). We further analyzed the KEGG enriched pathways [28] of genes in cluster 1, including *GADD45A* and *GADD45B*. The KEGG pathway revealed that *GADD45A* and *GADD45B* with the co-expression genes were mainly enriched in the TNF signaling pathway (*p* = 3.36 × 10^−7^), the MAPK signaling pathway (*p* = 2.68 × 10^−7^), the IL-17 signaling pathway (*p* = 3.18 × 10^−7^), transcriptional mis-regulation in cancer (*p* = 4.36 × 10^−7^), and the NF-κB signaling pathway (*p* = 5.28 × 10^−7^) (Figure 6B).

### 2.6. Association of GADD45A and GADD45B with Infiltrating Immune Cells and Functions in Renal IRI and Validation

**Figure 7:**
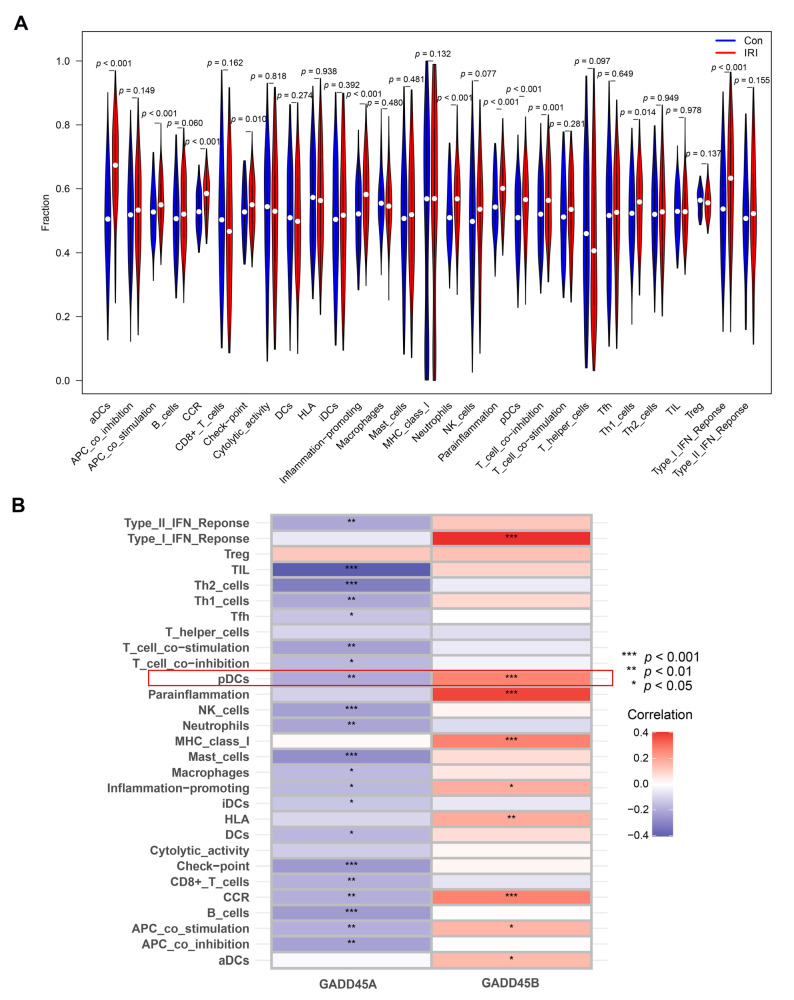
The association of *GADD45A* and *GADD45B* with infiltrating immune cells and functions using single-sample GSEA (ssGSEA) analysis in human renal ischemia-reperfusion injury (IRI). (**A**) Violin plot showing the fraction of infiltrating immune cells and functions in the renal IRI dataset (GSE43974). Fifteen types of immune cells and functions was significant difference. (**B**) The heatmap showing a total of 26 types of immune cells and functions had significant associations with *GADD45A* and *GADD45B* expression in renal IRI dataset. Treg, regulatory T cells; Th2 cells, T helper2 cells; Th1 cells, T helper1 cells; Tfh cell, Follicular helper T cell; pDCs, plasmacytoid dendritic cells; NK cells, natural killer cells; iDCs, immature dendritic cells; DCs, dendritic cells; aDCs, activated dendritic cells. Type I/II interferons (IFN) response, tumor-infiltrating lymphocytes (TIL), major histocompatibility complex (MHC) class I, inflammation-promoting, human leukocyte antigens (HLA), chemokine receptors (CCR), antigen-presenting cell (APC). * *p* < 0.05, ** *p* < 0.01, *** *p* < 0.001.

We further analyzed the relationship between the hub genes and 29 types of infiltrating immune cells and functions using an ssGSEA algorithm. The difference analysis in renal IRI was firstly performed. Eleven populations of immune cells (aDCs, neutrophils, pDCs, and Th1 cells) and functions (APC co-stimulation, check point, inflammation promotion, T cell co-inhibition, and Type I IFN response) were identified (Figure 7A) in the renal IRI dataset.

*GADD45A* showed a negative correlation with most types of immune cells and functions, while *GADD45B* showed a positive relationship with infiltrating immune cells and functions (Figure 7B). The expression of *GADD45A* was associated with a difference in the proportion of 13 populations of immune cells, including macrophages, B cells, and pDCs. *GADD45A* expression was also related to differences in immune cell function, including APC co-stimulation, T cell co-inhibition, and Type II IFN response. *GADD45B* expression was also associated with a difference in the proportion of pDCs and activated dendritic cells (aDCs).

**Figure 8:**
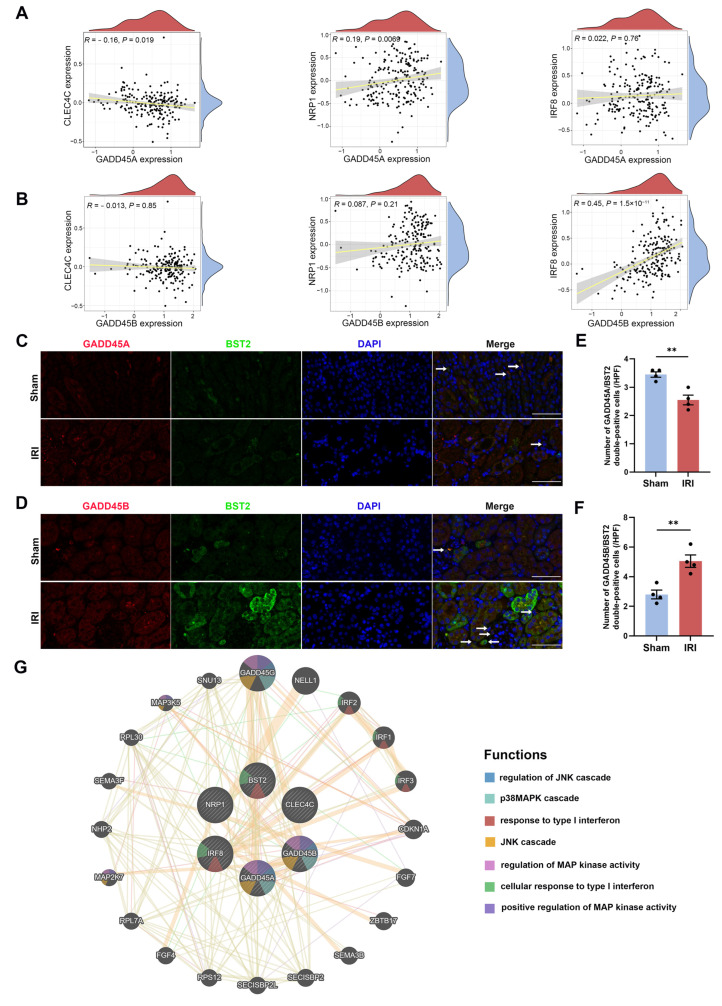
The correlation analysis of *GADD45A* and *GADD45B* expression with cell surface markers of plasmacytoid dendritic cells (pDCs) in human renal ischemia-reperfusion injury (IRI). (**A**) The significant relationship of *GADD45A* expression with cell surface markers (*CLEC4C* and *NRP1*) of pDCs. (**B**) The significant association of *GADD45B* expression with cell surface marker (*IRF8*) of pDCs. (**C**) The representative immunofluorescence pictures of GADD45A (red), BST2 (green), and DAPI (blue) are shown in sham-operated mice and renal IRI mice, GADD45A^+^BST2^+^ pDCs (white arrows), scale bars (white) = 50 μm. (**D**) The representative immunofluorescence pictures of GADD45B (red), BST2 (green), and DAPI (blue) are shown in sham-operated mice and renal IRI mice, GADD45B^+^BST2^+^ pDCs (white arrows), scale bars (white) = 50 μm. (**E**) The number of GADD45A^+^BST2^+^ and (**F**) GADD45B^+^BST2^+^ pDCs in the kidney tissue was counted, n = 4 per group. (**G**) The gene-to-gene network analysis of *GADD45A*, *GADD45B*, and pDC cell markers was constructed using the Metascape website and GeneMANIA online tool. MAPK pathways and type I interferon responses were identified. *CLEC4C*, C-Type Lectin Domain Family 4 Member C; *NRP1*, Neuropilin 1; *IRF*, Interferon Regulatory Factor 8; *BST2*, Bone Marrow Stromal Cell Antigen 2. ** *p* < 0.01.

As pDCs were the only intersecting immune cells of *GADD45A* and *GADD45B* expression, we further analyzed the association of *GADD45A* and *GADD45B* with the cell surface markers (*CLEC4C*, *NRP1*, and *IRF8*) of pDCs. While the *GADD45A* expression was inversely related to *CLEC4C* but positively related to *NRP1* expression (Figure 8A), *GADD45B* showed a positive correlation with *IRF8* expression (Figure 8B). To validate the association between pDCs and *GADD45A* and *GADD45B*, we performed immunofluorescence staining in the murine renal IRI model. The assay showed that the number of BST2 (a specific marker of pDCs) [29,30,31] and GADD45A double-positive infiltration pDCs decreased in renal IRI mice kidneys compared to the sham-operated mice (Figure 8C,E). Meanwhile, there was more GADD45B^+^BST2^+^ pDCs infiltration in the IRI group of mice (Figure 8D,F). The results were consistent with the ssGSEA analysis. Next, to further elucidate the putative molecular mechanisms of *GADD45A* and *GADD45B* in pDCs, a gene-to-gene network was constructed on the Metascape website [32] and GeneMANIA online tool [33]. MAPK pathways and type I interferon responses were identified, which were in concordance with the ssGSEA and KEGG pathway (Figure 8G).

### 2.7. GSEA and Immune Correlation Analysis of Low/High GADD45A and GADD45B Expression in Renal IRI

**Figure 9:**
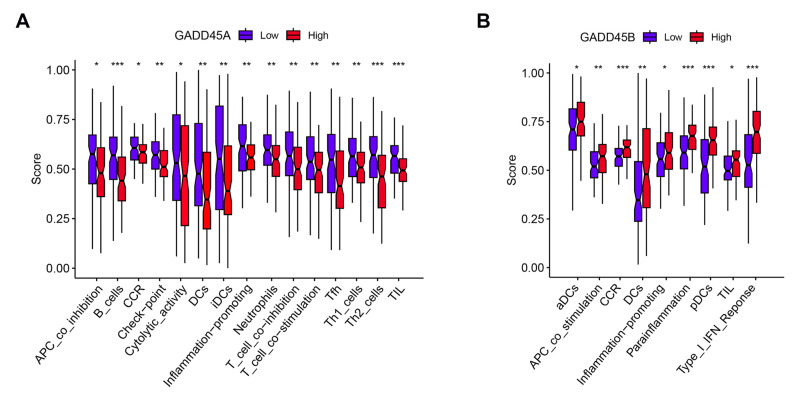
The immune correlation analysis of low/high *GADD45A* and *GADD45B* expression in human renal ischemia-reperfusion injury. (**A**) The significant influence of *GADD45A* expression in renal IRI based on immune cells and functions. (**B**) The significant influence of *GADD45B* expression in renal IRI based on immune cells and functions. * *p* < 0.05, ** *p* < 0.01, *** *p* < 0.001.

To gain further insight into the immune and functional role of *GADD45A* and *GADD45B* in renal IRI, the gene expression of *GADD45A* and *GADD45B* was divided into low- and high-expression groups according to the median gene expression in the renal IRI dataset. Low *GADD45A* expression was associated with increased immune cells infiltrating (B cells, DCs, iDCs) and enhanced immune functions (Figure 9A). The overexpression of *GADD45B* was significantly associated with higher DCs, iDCs, pDC infiltration and the expression of type I interferon response, and inflammation-promoting functions (Figure 9B). GSEA analysis revealed that *GADD45A* low-expression gene sets were associated with cell adhesion molecules, graft versus host disease, and ribosomes, while the high *GADD45A* expression gene set was mostly enriched on the metabolism (beta-alanine, drug cytochrome p450, and other enzymes), peroxisome, and valine leucine and isoleucine degradation (Figure S3A,B). Lower expression of *GADD45B* was related to the PPAR signaling pathway and metabolism (fatty acid, drug cytochrome p450 and xenobiotics by cytochrome), while *GADD45B* overexpression was related to the MAPK signaling pathway, NOD-like receptor signaling pathway, toll-like receptor signaling pathway, chemokine signaling pathway, and cytokine receptor interaction (Figure S3C,D).

### 2.8. Construction of Drug-mRNA-miRNA Network

**Figure 10:**
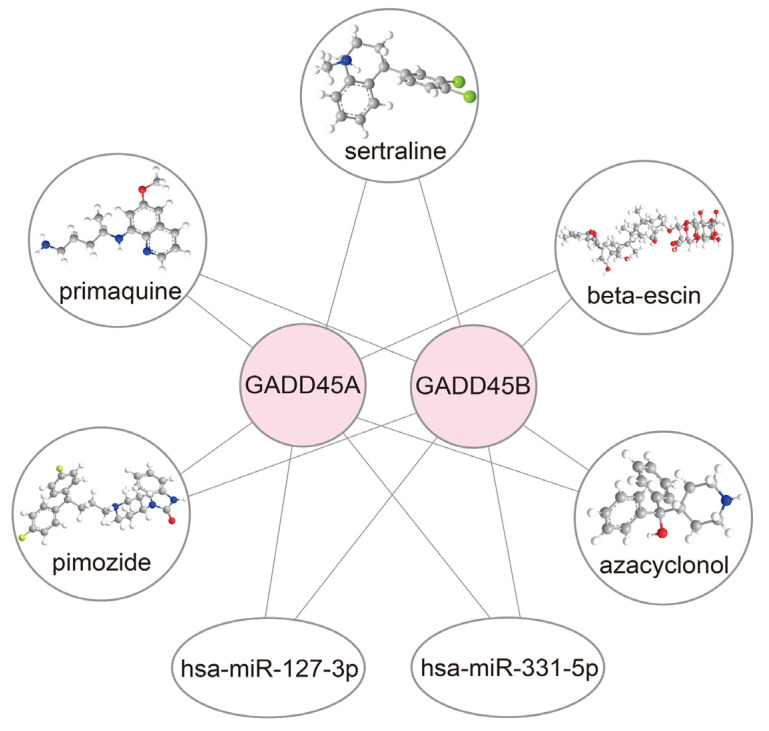
Interaction network showing the potential treatments for renal ischemia-reperfusion injury, *GADD45A*, *GADD45B*, and related microRNAs (miRs). The white circles represent the 3D structure tomographs of predicted drugs, while the white ovals represent the related miRs.

To develop potential treatments for renal IRI, we performed an interaction network analysis to identify drugs that target *GADD45A* and *GADD45B* and their relevant microRNAs (miRs). The candidate drugs that target *GADD45A* and *GADD45B* were selected through the DSigDB database [34]. The potential miRs targeting *GADD45A* and *GADD45B* were screened out from the TargetScan database [35]. A *p*-value of less than 0.05 was considered statistically significant. Therefore, the results from the DSigDB database suggested that *GADD45A* and *GADD45B* might be the potential targets of beta-escin, sertraline, primaquine, pimozide, and azacyclonol. In addition, hsa-miR-331-5p and hsa-miR-127-3p might be the promising interacting miRs of *GADD45A* and *GADD45B*, respectively (Figure 10).

## 3. Discussion

While accumulating evidence has suggested the roles of epigenetic alteration in renal IRI [12], the pathogenic significance of the upstream modulators of epigenetic changes, such as CRs, remains unclear. A recent bioinformatic analysis found that a high CR risk score was associated with adverse prognosis and immune cell infiltration, which was expected to be a therapeutic target for uterine corpus endometrial carcinoma [36]. Targeting the CR pathway could offer an intervention option and merits further attention. This study, to our knowledge, was the first to use a bioinformatic approach to systematically analyze the relationship between CRs, clinical outcomes, immune responses, and potential small-molecule drugs in renal IRI. Our results suggested that the dysregulation of CR-associated genes *GADD45A* and *GADD45B* was related to renal IRI and showed correlations with infiltrating pDCs. 

The pathophysiology of IRI remained elusive, and bioinformatics became an essential tool for discovering novel genes of IRI, such as renal IRI [37], cardiac IRI [38], hepatic IRI [39], and intestinal IRI [40]. In this study, we identified five CR-associated DEGs, including *DUSP1*, *GADD45A*, *GADD45B*, *GADD45G*, and *HSPA1A*, which were upregulated in human renal IRI tissues after kidney transplantation. Further GO and KEGG pathway enrichment analyses suggested that these CRs were involved in cell cycles, p38 MAPK, p53, FoxO, and NF-κB signaling pathways. Recently, two bioinformatic studies have been published in which renal IRI biomarkers were enriched in the MAPK pathway [41,42]. Although these bioinformatical data were derived from animal experiments, they were in line with our findings from human renal IRI and further confirmed the importance of CRs and the MAPK pathway in renal IRI. Indeed, these pathways were highly relevant to the regulation of immune cell function [21], inflammation [43], cell cycle arrest [44], autophagy [45], and apoptosis [46], suggesting the essential roles of CRs in renal IRI and subsequent kidney repair. Among these CR-associated DEGs, the top two genes (*GADD45A* and *GADD45B*) were screened through the CytoHubba plugin in Cytoscape [25]. These two hub genes are more likely to play a role in biological regulation in renal IRI. To enhance the clinical relevance of our data, *GADD45A* and *GADD45B* were selected to establish a predictive model for renal IRI and performed the best predictive value among all gene combinations. This risk model, validated also by external datasets from GEO, demonstrated good performance for predicting renal IRI. Data from our animal studies, which showed significant upregulation of *GADD45A* and *GADD45B* in mice kidneys after renal IRI, also corroborated our findings from bioinformatics analyses.

The growth arrest and DNA damage-inducible 45 (GADD45) family proteins are composed of GADD45A, GADD45B, and GADD45G, which are the crucial sensors for cell homeostasis and survival in response to various stressors [47]. Mammalian GADD45 proteins are broadly expressed in kidneys, heart, brain, liver, testis, and skeletal muscle [48], where they carry out diverse functions, including regulating stress response, apoptosis, DNA demethylation, DNA repair, cell differentiation, embryonic regulation, as well as cell growth and aging [47,49,50]. GADD45 family proteins function together with interacting proteins, such as p38, c-Jun N-terminal kinase, proliferating cell nuclear antigen, and p21 [49]. Previous research found that the pathogenesis of renal IRI has been linked to oxidative stress [8], apoptosis [11], and mitochondrial [8] and DNA damage [9]. Therefore, it is reasonable to believe that GADD45 proteins are involved in diverse biological and pathological processes of renal IRI. Our findings of upregulated GADD45A and GADD45B expressions in renal IRI samples were in line with the results in other types of IRI. Previous studies have reported increased GADD45A expression in the peripheral blood samples of patients with myocardial IRI and in rat heart tissues after left main coronary artery ischemia and reperfusion [51,52]. Furthermore, the binding of miR-1283 to the 3′ untranslated region of GADD45A could protect against hypoxia/reoxygenation-induced human embryonic cardiomyocytes apoptosis via the p38 MAPK signaling pathway [53]. GADD45A was also upregulated in mice hippocampus after cerebral IRI [54]. The downregulation of HECT, UBA, and WWE domain-containing 1 (Huwe1) and upregulation of GADD45A conferred neuroprotection during ischemia and reperfusion, which may be through the Huwe1-p53-GADD45A axis [55]. Increased GADD45A expression was also observed in liver IRI, and this has been proposed as a marker of hepatic IRI [56,57]. Moreover, GADD45A is also involved in many key processes of kidney diseases, such as cell cycle progression in CKD [58], DNA damage or repair in doxorubicin-induced kidney injury [59], and resistance to chemotherapy or radiotherapy in renal cell carcinoma cells [60,61]. The dysregulation of the other hub gene—*GADD45B*—has been implicated largely in conditions related to IRI in the brain. GADD45B was upregulated in ischemic stroke and could attenuate cerebral ischemia-induced neuronal apoptotic death and axonal plasticity [62,63,64]. Reduced GADD45B expression induced depression-like behaviors after cerebral ischemia by releasing pro-inflammatory cytokines [65]. The overexpression of activin receptor-like kinase 5 (ALK5) mediated neural plasticity and neurological function recovery after cerebral IRI by targeting GADD45B expression [66]. Data from in vitro studies also suggested that GADD45B could protect against rat primary cortex neurons under oxygen-glucose deprivation and reperfusion via the inhibition of autophagy and apoptosis [67]. In addition, GADD45B was upregulated in diabetic mice with myocardial IRI [68]. Similar to GADD45A, GADD45B expression was increased in various glomerular diseases, such as IgA nephropathy [69] and diabetic kidney disease [70]. While these observations suggest that GADD45A and GADD45B may play important roles in renal IRI and regulate cellular functions in a pathology- and tissue-dependent manner, the exact molecular mechanisms remain to be elucidated. In our study, the MAPK pathway was involved in all the enriched analysis of CR-related DEGs identified by machine learning, co-expression genes with *GADD45A* and *GADD45B* selected by WGCNA, and significant pDC cell markers with *GADD45A* and *GADD45B*. While these findings all indicate that *GADD45A* and *GADD45B* may play crucial pathogenic roles in the MAPK pathway, our results need to be further verified with basic/animal experiments and clinical samples from patients with renal IRI.

The activation of resident immune cells and the adhesion and infiltration of circulating leukocytes are crucial immunological events in renal IRI [21]. It is recognized that epigenetic regulation can orchestrate the differentiation and response to stimuli of immune-reactive cells [71]; hence, it would be worthwhile to investigate how upstream regulators such as CRs affect infiltrating immune cells in renal IRI. Our results showed that the abnormal expression of *GADD45A* and *GADD45B* was associated with altered proportions of distinct immune cell populations, including pDCs, macrophages, neutrophils, and T helper cells. Among these important immune cell types, we found that pDCs were the only intersection immune cells related to *GADD45A* and *GADD45B* expression, and the association between *GADD45A*, *GADD45B*, and the key cell surface markers (*CLEC4C*, *NRP1*, and *IRF8*) of pDCs lent further support to our observations. pDCs is a unique DC subset that specializes in antigen presentations and the production of type I IFN and other inflammatory cytokines/chemokines in response to viral infections [72]. Mounting evidence has suggested the pathogenic contribution of pDCs in the IRI of various organs. The rapid infiltration of pDCs into the kidney has been observed after AKI induced by renal IRI or cisplatin toxicity, and the depletion of pDCs attenuated kidney damage, while the adoptive transfer of pDCs aggravated it [73]. Moreover, IFN-α produced by pDCs plays a detrimental role in renal IRI-induced AKI models and in AKI patients after kidney transplantation [73]. High proportions of activated pDCs and IFN-mediated organ insults were also observed in hepatic IRI [74] and myocardial IRI [75]. The roles of *GADD45A* and *GADD45B* in immune regulation have also been reported in previous studies. The deletion of *GADD45A* and *GADD45B* resulted in disturbed cellular functions in granulocytes and macrophages [76]. Furthermore, *GADD45B* also mediates the protective effect of *CD40* against Fas-induced apoptosis in B lymphocytes [77]. In the synovial fluid of rheumatoid arthritis, high *GADD45B* expression enhances Th1 cell survival [78]. Our results found a negative association between *GADD45A* and pDCs and a positive association between *GADD45B* and pDCs. Previous research found that the p38 MAPK pathway activation contributes to the INF-α expression of pDCs [79]. *GADD45B* has been demonstrated to play an essential role in the maintenance of the p38 MAPK pathway activation [80]. In contrast, in *GADD45A*-deficient mice, the p38 MAPK pathway activation occurred. It was further confirmed that recombinant *GADD45A* could suppress the p38 MAPK pathway activation [81]. Therefore, it is speculated that *GADD45A* and *GADD45B* exert different immunity regulation functions through the p38 MAPK pathway. This needs further validation in future studies.

As our findings illustrate a close relationship between *GADD45A* and *GADD45B* in renal IRI, drugs that target *GADD45A* and *GADD45A* can become potential treatment to prevent or ameliorate renal IRI. Here, we identified that beta-escin, sertraline, primaquine, pimozide, and azacyclonol are drugs that target *GADD45A* and *GADD45B*. Beta-escin is the main active component of escin, a mixture of saponins extracted from *Aesculus hippocastanum* (horse chestnut), and it shows anti-oxidative, anti-inflammatory, and vasoprotective effects. Escin could protect against oxidative stress and decrease inflammatory factors expression in chronic MPTP/probenecid mouse models of Parkinson’s disease [82]. Sertraline is a selective serotonin receptor inhibitor commonly used as an anti-depressant, and accumulating data show that sertraline could reduce neuroinflammation and oxidative stress in experimental models of depression [83]. Primaquine is an antimicrobial used for the treatment of malaria and pneumocystis jiroveci. While our finding that primaquine may protect against renal IRI appears counterintuitive, as this drug is known to induce oxidative stress, it remains possible that its beneficial effects may derive from its anti-inflammatory actions [84]. Pimozide is a diphenylbutylpiperidine that can be used to treat schizophrenia, Tourette’s syndrome, and recurrent tic [85]. The pretreatment of mice with pimozide attenuated the cecal ligation puncture-responsive induction of proinflammatory cytokines in the liver and interstitial edema in the lung in a murine sepsis model, via the inhibition of FABP-4 [86]. Azacyclonol is an old anti-psychotic drug that was discontinued due to poor and mixed clinical effectiveness in schizophrenia [87]; hence, whether to re-explore the use of this agent in other conditions, such as renal IRI, remains questionable. Our findings are clinically relevant because they provide scientific rationales for repurposing these drugs for the management of renal IRI. To further understand the pharmacological mechanisms of these drugs, we also elucidated the relationship between these drugs, *GADD45A*, *GADD45B*, and related miRs. Here, we predicted that hsa-miR-331-5p and hsa-miR-127-3p may affect *GADD45A* and *GADD45B* at a post-transcriptional level. Indeed, previous studies have suggested that miR-127-3p was regulated by HIF-1α after renal IRI in rat IRI models [88], and altered miR-127-3p expression showed a diagnostic value in patients with AKI [89]. While there are limited data on the correlation between hsa-miR-331-5p and renal IRI, the abnormal expression of hsa-miR-331-5p has been reported in different ischemic conditions. For instance, miR-331-5p overexpression was related to the activation of cerebral ischemia caused by inflammasome [90]. A recent study also suggested that hsa-miR-331-5p might be a novel target for the treatment of perinatal asphyxia and hypoxic-ischemic encephalopathy [91]. Because a single miRNA achieves its function by targeting multiple downstream genes, targeting miR-127-3p or miR-331-5p may fulfill divergent biological roles in renal IRI. Legumain (*LGMN*) is one of the target genes of miR-127-3p [92]. The miR-127-3p-mediated repression of *LGMN* mRNA may attenuate the autophagy of GPX4 and ferroptosis in renal tubular epithelial cells, therefore protecting renal IRI [93]. In addition, tumor necrosis factor receptor-associated factor 6, a target gene of miR-331-5p [90], is involved in inflammation and oxidative stress in renal IRI [94]. The regulation of miR-331-5p may functionally ameliorate renal IRI-induced inflammation and oxidative stress.

One important limitation of our study was that the utility of targeting of *GADD45A* or *GADD45B* for therapeutic benefit should be systematically validated by genetic approaches, such as knockout animal model and knockdown cell line strategies. Notwithstanding, we measured the intra-renal expression of *GADD45A* and *GADD45B* in a murine renal IRI model to validate our bioinformatic results. Additionally, we did not administer the drugs that target *GADD45A*/*GADD45B* in mice to examine their protective effects on renal IRI. While our present bioinformatic results were largely generated from secondary analyses of data obtained from public domains, such data were acquired from different datasets, which ensured better validity and impartiality. The correlations between pDCs, *GADD45A*, and *GADD45B* will require clinical validation. Further clinical and translational studies will be required to verify our current bioinformatics/animal findings and evaluate our risk prediction model and the repurposing of drugs identified in this study. Nevertheless, we also conducted IF (Figure 8C,D), IHC (Figure 4H), and Western blot (Figure 4I) experiments to validate our bioinformatic analysis data.

## 4. Materials and Methods

### 4.1. Datasets Collection

Human datasets related to renal IRI in the Gene Expression Omnibus (GEO) (https://www.ncbi.nlm.nih.gov/geo/ (accessed on 27 April 2022)) database were all selected. We further chose the number of cases, which was over 50. Only one dataset, GSE43974, fit the requirement. Thus, we merged the other two human datasets for validation. The human kidney transplant tissues dataset (GSE43974) included 203 renal IRI cases and 188 control samples [95]. The dataset was first annotated using the platform of GPL10558. Next, we matched the probe to their gene symbols using “impute”, “limma” R language. Eight hundred and seventy chromatin regulator-related genes were selected from a previous publication [13]. Two human kidney injury tissues datasets—GSE30718 (11 control samples and 28 IRI cases) [96] and GSE126805 (41 control samples and 42 IRI cases) [97]—were merged and used to validate the gene expression and the predictive renal IRI risk model.

### 4.2. Identification of Chromatin Regulators-Associated Differentially Expressed Genes

Differentially expressed genes (DEGs) related to CRs were identified by the “limma” R package (accessed on 7 May 2022) [98]. Genes with the filter of *p*-value < 0.05 and |log2 Fold change (FC)| ≥ 0.5 were regarded as DEGs using the “limma” R package (https://bioconductor.org/packages/release/bioc/html/limma.html (accessed on 7 May 2022)).

### 4.3. Identification CR-Related Characteristic Biomarkers Using Machine Learning Approach

To screen out the important CR-related DEGs, three machine learning models, including random forest (RF) [23], support vector machine (SVM) [22], and extreme gradient boosting (XGB) [24], were constructed. The associated R packages were “randomForest” [23], “xgboost” [24], and “kernlab” [99]; the “pROC” [100] package was used to draw the receiver operator characteristic (ROC) curves and assess the accuracy.

### 4.4. Gene Ontology (GO), Kyoto Encyclopedia of Genes and Genomes (KEGG) Enrichment Analysis and Gene Set Enrichment Analysis

GO and KEGG enrichment analyses were carried out via the R package “ClusterProfiler” [101], “RColorBrewer”, and “enrichplot”. Both *p* and *q* values of less than 0.05 were considered meaningful. The gene set enrichment analysis (GSEA) enriched pathway analysis was further performed by “clusterProfiler”, “enrichplot”, “org.Hs.eg.db”, and the “limma” R package based on the “c2.cp.kegg.Hs.symbols.gmt” gene set, with a threshold of *p* < 0.05.

### 4.5. Protein-Protein Interaction (PPI) Network Construction

The PPI network was constructed by the online STRING (Search Tool for the Retrieval of Interacting Genes/Proteins) tool (https://string-db.org/ (accessed on 9 May 2022)) [102]. This database combines the interactions between protein and protein through physical and functional associations, integrating a large amount of information from many reliable resources, such as BioGRID, OMIM, Gene Ontology, and GEO database. Interactions could be evaluated and scored. In this study, we firstly put the gene list of CR-associated DEGs in renal IRI in the STRING tool [102] and chose the advanced setting of the minimum required interaction score with medium confidence (0.400). The visualization of the PPI network was constructed using Cytoscape software (version 3.9.1). 

Another PPI network of co-expression genes with GADD45A and GADD45B was also constructed using the STRING tool [102]. The genes were put into the gene list using the online STRING tool at the threshold of the 0.700 minimum confidence (high confidence) and hiding the disconnected nodes. The network was divided into three clusters according to the k-means algorithms.

To predict the potential pathways in renal IRI pDCs, the relationship of hub genes (GADD45A and GADD45B) and pDC cell markers (CLEC4C, NRP1, IRF8, BST2) was examined using GeneMANIA [33].

### 4.6. Screening the Co-Expression Genes Using WGCNA Network Analysis

To obtain more insight into the hub genes associated underlying mechanisms in renal IRI, we performed a weighted correlation network analysis (WGCNA) [101,103] to select the co-expression genes. We firstly detected the 25% upper variation and removed the out-liner renal IRI samples in the GSE43974 dataset. The data were merged to construct the gene co-expression network. The soft thresholding power β was calculated to construct adjacency. The adjacency was transformed and assessed using a topological overlap matrix. We finally used the β = 8 (scale free R2 = 0.85) as the soft threshold. Next, the hierarchical cluster and dynamic tree cut identified the 10 modules based on topological overlap matrix-related dissimilarity calculation, with at least 50 genes the size of a dendrogram. The relationship between module membership and gene significance was analyzed. The most significant module was chosen, and its intersection genes with DEGs in the renal IRI dataset were selected for the subsequent analysis using the EVenn website (http://www.ehbio.com/test/venn/#/ (accessed on 18 April 2023)) [104].

### 4.7. Analysis of Infiltrating Immune Cells

The single-sample gene set enrichment analysis (ssGSEA) was further utilized to accurately and reliably assess the infiltration score of 29 types of immune cells and immune functions in each renal IRI patient based on the “GSVA” R package. These immune cells included Treg, Th2 cells, Th1 cells, Tfh cells, pDCs, NK cells, neutrophils, mast cells, macrophages, iDCs, DCs, CD8^+^ T cells, B cells, and aDCs. The immune functions analyzed included type I/II IFN response, TIL, T cell co-stimulation/inhibition, para-inflammation, MHC class I, inflammation-promoting, HLA, cytolytic activity, chemokine receptors, and APC co-stimulation/inhibition.

The relationships of hub genes with infiltering immune cells were further examined using “limma”, “reshape2”, “tidyverse”, and “ggplot2” R packages. The significant alteration in immune cell fractions was identified according to the Wilcoxon test threshold of *p*-value < 0.05. Associations between different immune cell subtypes were evaluated using the Pearson correlation coefficient method.

### 4.8. Evaluation of Relationship between Hub Genes and Cell Surface Markers of Plasmacytoid Dendritic Cells

The relationship between the hub genes and cell surface markers of plasmacytoid dendritic cells (pDCs) was further explored using the R “ggExtra” and “vioplot” pack-age. The cell surface markers of pDCs used for analysis were according to the CellMarker website (http://bio-bigdata.hrbmu.edu.cn/CellMarker/ (accessed on 12 June 2022)).

### 4.9. Construction of a CRs-Associated Risk Predictive Model for Renal Ischemia-Reperfusion Injury

LASSO-Cox regression algorithm was employed to construct the risk predictive model using “rms”, “enrichplot”, and “ROCR” R packages. The risk scores of the IRI calculation formula were as follows:

$$\mathrm{Risk}\;\mathrm{scores}=\sum_{i=1}^{n}\left(Expression\;mRNAi\times Coefi\right)$$

The levels of expression were divided into high- or low-expression groups based on the median mRNA expression. ROC curves were constructed to assess the performance of the renal IRI risk score model. The levels of expression were divided into high- or low-expression groups based on the median mRNA expression. In the nomogram, the points were calculated according to gene expression, and the total point was used to predict the risk of renal IRI development. It was a robust predictive model that has been used in other publications [105,106].

### 4.10. Validation of Bioinformatics Data by Murine Renal IRI Model

To validate the findings of our bioinformatics data, we used a murine renal IRI model to evaluate the expression of CR-related genes. Ten-week-old male C57BL/6 mice (weight, 23–25 g) were housed under controlled temperature and humidity conditions, with a 12-h light-dark cycle, and they received food and water ad libitum. All experimental procedures were approved by the local Animal Care and Use Committee of the Nanfang Hospital of Southern Medical University. For experiments, mice were divided into two groups (Sham and IRI group), each with 8 mice. Renal function markers, kidney histopathology, and *GADD45A*/*GADD45B* mRNA levels were evaluated in all mice, whereas immunohistochemistry and Western blot assay were evaluated in 6 mice in each group. Immunofluorescence staining was evaluated in 4 mice in each group. After one week of acclimatization, mice were anesthetized with pentobarbital sodium (60 mg/kg intraperitoneal), and the body temperature was maintained at 37 °C using a heating pad. Renal pedicles were exposed by an abdominal midline incision, followed by bilateral clamping to induce ischemia for 35 min. The clips were then released to allow reperfusion. Sham-operated mice underwent the same procedure without clamping. At 24 h after reperfusion, the kidney samples were harvested for further processing [107]. Blood was also collected for serum creatinine (SCr) and blood urea nitrogen (BUN) measurement using a Creatinine Assay kit (C011-2-1, Nanjing Jiancheng) and Urea Assay Kit (C013-2-1, Nanjing Jiancheng) respectively. 

### 4.11. Validation of Hub Genes and Kidney Injury Biomarkers Expression Using Quantitative Real-Time Polymerase Chain Reaction (qRT-PCR)

The total RNA of genes was extracted from the mouse kidney tissues using Trizol reagent (Invitrogen Life Technologies, Carlsbad, CA, USA) according to the previous protocol [108]. Complementary DNA synthesis was carried out by the ReverTra Ace qPCR RT Kit (FSQ-101, TOYOBO, Osaka, Japan) according to the manufacturer’s instructions. SYBR Green PCR Master Mix (QPK-201, TOYOBO, Osaka, Japan) was further used to perform qRT-PCR. We analyzed the transcriptional level of hub genes by the 2^−ΔΔCt^ method. The primer sequences for qRT-PCR were as follows (Table 2).

### 4.12. Renal Histopathology

Formalin-fixed kidney tissues were embedded in paraffin. The fixed renal tissues were cut into 4 µm thick sections and stained with periodic acid-Schiff (PAS) reagents. The histological examination was performed in a blinded manner, using 10 randomly selected and non-overlapping fields (400×). Tubular damage scores were determined according to the percentage of necrosis and detachment of tubular epithelial cells, the formation of protein cast, and the disappearance of the brush border in the corticomedullary region (score 0 = 0%; score 1 = 1% to 10%; score 2 = 11% to 25%; score 3 = 26% to 50%; score 4 = 51% to 75%; score 5 = 76% to 100%) [109].

### 4.13. Candidate Drugs and Microrna-Targeted Screening

We further screened the candidate drugs from the DSigDB [34] database and the microRNA (miRNA) targeting gene from the TargetScan database (https://maayanlab.cloud/Enrichr/ (accessed on 29 May 2022)). A *p*-value of less than 0.05 was considered statistically significant. The network of the drugs-mRNA-miRNA target gene was further constructed using Cytoscape software (version 3.9.1), and the 3D structures of drugs were displayed using the PubChem website [110] (https://pubchem.ncbi.nlm.nih.gov/ (accessed on 29 May 2022)) and ChemBio3D Ultra 14.0 [111].

### 4.14. Western Blot Analysis

The protein of mouse kidney tissues was extracted using RIPA buffer (PC101, Epizyme Biotech, Shanghai, China) supplemented with protease inhibitor, and the protein concentration was determined using the BCA protein assay kit (P0010, Beyotime Biotechnology, Shanghai, China). A total of 32 μg protein was separated by 10% SDS/PAGE and transferred to the PVDF membrane. PVDF membranes were blocked with Rapid Blocking Buffer (PS108, Epizyme Biotech, China) for 1 h and incubated at 4 °C overnight with the primary antibody, including anti-GADD45A (1:1000, bs-1360R, Bioss, Beijing, China), anti-GADD45B (1:1000, bs-15904R, Bioss, Beijing, China), and anti-β-actin (1:1000, 4970, Cell Signaling Technology, Danvers, MA, USA). The following day, PVDF membranes were incubated with a goat anti-rabbit secondary antibody for 1 h at room temperature. Finally, the proteins were visualized by the ECL Enhanced system [108].

### 4.15. Immunohistochemistry (IHC) and Immunofluorescence (IF) Staining

IHC staining was performed as previously described [112]. Paraffin-embedded mouse kidney specimens were cut into 3 μm thick sections, and then the samples were deparaffinized in xylene and rehydrated in graded ethanol. Endogenous peroxidase in kidney tissue was blocked with 3% hydrogen peroxide. After the non-specific binding was blocked with normal goat serum (10%), the slides were incubated with anti-GADD45A (1:500, bs-1360R, Bioss) and anti-GADD45B (1:500, bs-15904R, Bioss) at 4 °C overnight. After secondary antibody incubation, the slices were stained using the DAB kit (Dako, Carpinteria, CA, USA), and hematoxylin counterstaining was then performed.

Fresh mice kidneys were dehydrated, embedded, and frozen into 3 μm thick slices. The slides were incubated with primary antibodies against GADD45A (1:200, bs-1360R, Bioss), GADD45B (1:200, bs-15904R, Bioss), and BST2 (CD317, PDCA-1, 1:100, 13560-1-AP, Proteintech) overnight at 4 °C. Then the slides were washed using phosphate-buffered saline 3 times, and the second antibodies were added for 50 min in darkness at room temperature. Finally, the slides were incubated with DAPI (AR1176, BOSTER, Wuhan, China) in darkness for 10 min at room temperature for cell nuclear staining. The slides were observed using a fluorescence microscope [108].

### 4.16. Statistical Analysis

All statistical analyses were performed using R software (version 4.1.3) and GraphPad Prism software (https://www.graphpad.com/ (accessed on 31 July 2022), version 9.4.0). The difference between the groups was analyzed by Student’s *t*-test (data with normal distribution) and Wilcoxon rank-sum test (data with non-normal distribution) as appropriate. All data were presented as the mean ± standard error of mean. A threshold of *p* < 0.05 was considered statistically significant.

## 5. Conclusions

The dysregulation of CRs, especially *GADD45A* and *GADD45B*, is related to renal IRI and the infiltration of distinct immune cells. These differentially expressed CRs hold promise for prognostication and treatment in renal IRI.

## Figures and Tables

**Table 1:** Gene ontology analysis of five chromatin regulator (CR)-related differentially expressed genes.

ID	Description
GO:1900744	regulation of p38 MAPK cascade
GO:0038066	p38 MAPK cascade
GO:1900745	positive regulation of p38 MAPK cascade
GO:0032872	regulation of stress-activated MAPK cascade
GO:0070302	regulation of stress-activated protein kinase signaling cascade
GO:0051403	stress-activated MAPK cascade
GO:0016607	nuclear speck
GO:0047485	protein N-terminus binding

**Table 2:** The primer sequences of qRT-PCR.

Gene	Forward Primer (5′–3′)	Reverse Primer (5′–3′)
*18S*	CGATCCGAGGGCCTCACTA	AGTCCCTGCCCTTTGTACACA
*NGAL*	GCCTCAAGGACGACAACATC	CTGAACCATTGGGTCTCTGC
*KIM-1*	TTGCCTTCCGTGTCTCTAAG	AGATGTTGTCTTCAGCTCGG
*GADD45A*	CCGAAAGGATGGACACGGTG	TTATCGGGGTCTACGTTGAGC
*GADD45B*	GCCAAACTGATGAATGTGGACC	GAACGACTGGATCAGGGTGA

## Data Availability

The datasets presented in this study can be found in online repositories. The names of the repository/repositories and accession number(s) can be found in the article. The rest of the data are available from the corresponding author upon reasonable request.

## Supplementary Materials

The supporting information can be downloaded at: https://www.mdpi.com/article/10.3390/ijms241411304/s1.
