# Supplementary material for: GADD45A and GADD45B as Novel Biomarkers Associated with Chromatin Regulators in Renal Ischemia-Reperfusion Injury
Source: Int J Mol Sci. 2023 Jul 11;24(14):11304. doi: 10.3390/ijms241411304 (PMC10379085; doi:10.3390/ijms241411304)
Supplement: Supplementary file 1 [file ijms-24-11304-s001.zip › Supplemental figures.pdf]

Supplementary Figure 1

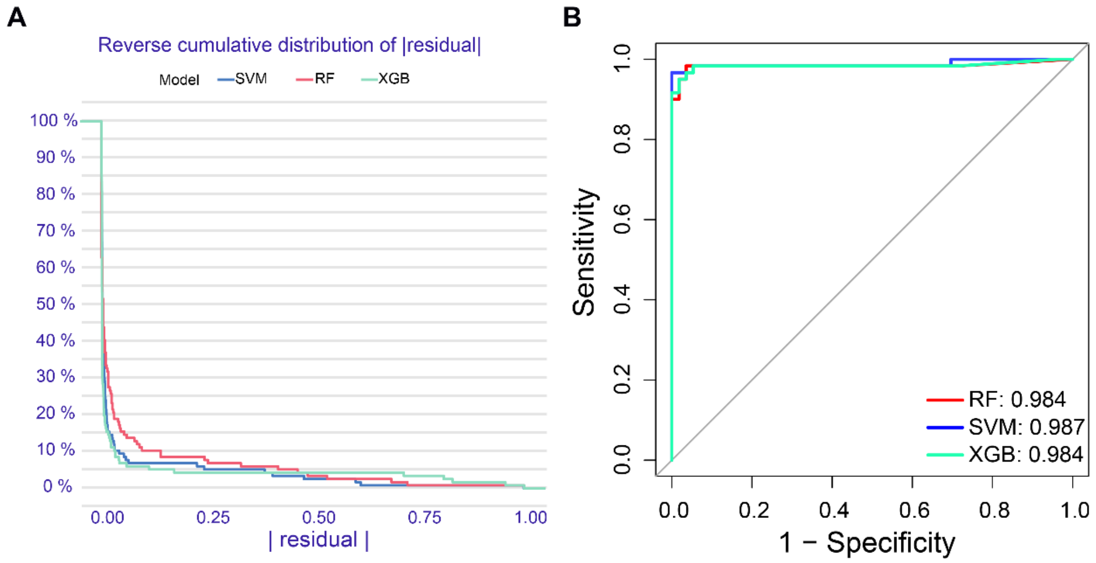

Figure S1. Three machine learning models in human renal ischemia-reperfusion injury dataset. (**A**) Reverse cumulative distribution of the residual and (**B**) Receiver Operating Characteristic curves of the SVM (Support Vector Machine), RF (Random Forest) and XGB (Extreme Gradient Boosting) machine learning models.

Supplementary Figure 2

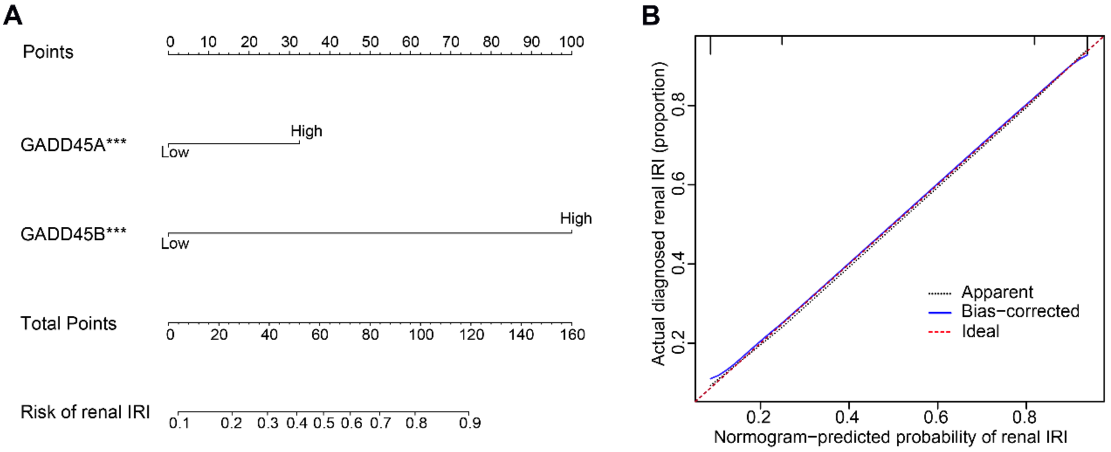

Figure S2. The predictive nomogram and calibration curve in human renal ischemia-reperfusion injury dataset. (**A**) The nomogram of *GADD45A* and *GADD45B* low-/high-gene expression score in the GSE43974 dataset (training cohort). (**B**) Nomogram calibration curve in the training cohort. *GADD45A* (Growth Arrest and DNA Damage Inducible protein 45 alpha); *GADD45B* (Growth Arrest and DNA Damage Inducible protein 45 beta). *** $p < 0.001$.

Supplementary Figure 3

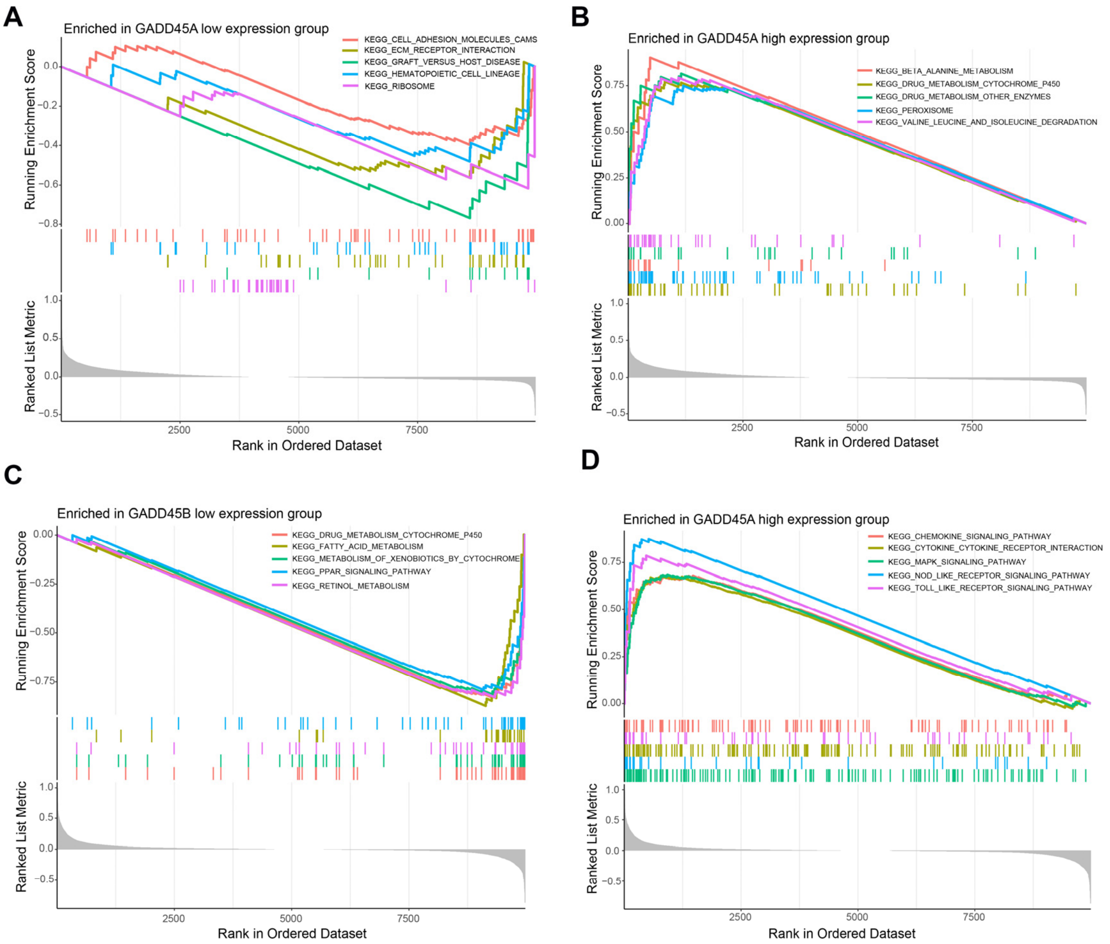

Figure S3. Gene set enrichment analysis (GSEA) of low/high *GADD45A* and *GADD45B* expression in human renal ischemia-reperfusion injury. (**A**) The Kyoto Encyclopedia of Genes and Genomes (KEGG)-enriched low-*GADD45A* gene sets using the GSEA method. (**B**) The KEGG-enriched high-*GADD45A* gene sets using the GSEA method. (**C**) The KEGG-enriched low-*GADD45B* gene sets using GSEA method. (**D**) The KEGG-enriched high-*GADD45B* gene sets using the GSEA method.
